# Immersive virtual reality for chronic neuropathic pain after spinal cord injury: a pilot, randomized, controlled trial

**DOI:** 10.1097/PR9.0000000000001173

**Published:** 2024-10-08

**Authors:** Laura Tabacof, Sophia I. Salazar, Erica Breyman, Leila Nasr, Sophie Dewill, Annie Aitken, Alexandra Canori, Michael Kypros, Mar Cortes, Adam Fry, Jamie Wood, David Putrino

**Affiliations:** Department of Rehabilitation and Human Performance, Icahn School of Medicine at Mount Sinai, New York, NY, USA

**Keywords:** Neuropathic pain, Spinal cord injury, Virtual reality, Chronic pain, Nonpharmacological intervention, Neuromodulation, Pain management

## Abstract

**Introduction:**

Neuropathic pain (NP) poses significant challenges for individuals with spinal cord injury (SCI), often inadequately managed by current interventions. Immersive virtual reality (IVR) has emerged as a promising approach for pain modulation, yet robust evidence is lacking.

**Objectives:**

This pilot study investigated the analgesic effects of different IVR environments (scenic, somatic) compared with a control environment, and explored psychomotor properties influencing pain attenuation.

**Methods:**

Twenty-two participants with NP caused by SCI were randomized into 3 IVR environments: somatic (n = 8), scenic (n = 7), and control (n = 8), undergoing 3 weekly sessions over 4 weeks with baseline, postintervention, and one-month follow-ups.

**Results:**

There was a significant interaction effect between VR environment and time point on Neuropathic Pain Symptom Inventory scores (F(4,37.0) = 2.80, P = 0.04). Scenic VR participants exhibited reduced scores postintervention and at follow-up, with no significant changes in somatic VR or control environments. Similar trends were observed in secondary measures, such as Neuropathic Pain Scale and pain numeric rating scale. Enjoyment and presence were associated with decreased pain-change scores (F(1, 252) = 4.68, P = 0.03 for enjoyment; F(1, 223.342) = 7.92, P = 0.005 for presence), although not significantly influenced by VR environment or time point.

**Conclusion:**

Both environments showed reduced pain outcomes, underscoring the need for personalized IVR pain therapies and informing further technology development for NP management.

## Abstract 2

Immersive virtual environments as a nonpharmacological pain intervention is associated with significant pain reductions and improvements in function and quality of life, compared with control.

## 1. Introduction

Spinal cord injury (SCI) affects more than 27 million people globally, causing an estimated 9.5 million years of healthy life lost because of disability.^21^ Chronic pain often co-occurs with SCI, significantly affecting quality of life.^52^ Neuropathic pain (NP), with an estimated prevalence of 65% postinjury, is a particularly disruptive secondary health conditions for people with SCI.^1,3,49^

Neuropathic pain, occurring at or below the level of SCI, involves maladaptive responses of the somatosensory nervous system.^12,13^ In SCI with sensory deafferentation, cortical and subcortical sensory maps become reorganized and lead to the brain interpreting aberrant nociceptive impulses from the spinal somatosensory circuit as pain.^13,15,27,29^ Standard pharmacological interventions, such as analgesics, antidepressants, and anticonvulsants/antiepileptics, are known to insufficiently manage pain.^4,10,23,34^ Alternatively, nonpharmacological interventions, including transcranial direct current stimulation and transcutaneous electrical nerve stimulation, show promise but are often inaccessible due to cost or availability.^16,20,28,42,54^ Thus, alternative nonpharmacological approaches that are accessible and pose minimal risk to patients are critically needed.

Virtual reality (VR) has emerged as a potential neuromodulation-based intervention for pain with minor side effects.^3,17,22,25,33,46,51,56^ Immersive virtual reality (IVR) uses a head-mounted display, enabling users to interact with a 3-dimensional virtual environment, fostering immersion, presence, and embodiment.^17,43,50^ Presence, in a virtual environment (VE), results from integrating 2 factors: place illusion and plausibility of content.^52^ Presence in a VE is known to have a powerful distraction effect, crucial for acute pain modulation.^25^ Embodiment refers to the illusion of owning virtual limbs or a body, which can be therapeutically manipulated to address chronic pain.

Immersive virtual reality is thought to produce an analgesic effect through 2 mechanisms: distraction or embodiment. Distraction mechanisms have been widely described, mostly for acute pain, and explained by the gate control theory of pain and the upregulation of nonpainful neural signaling/downregulation of nociceptive signals.^5,22,37,40,45^ Conversely, embodiment mechanisms follow the rationale posed by Moseley et al. , that is, manipulation of body image through embodiment in VR targets the somatosensory cortical map correction, akin to Mirror Box Therapy modalities.^35,38,48^ In addition, psychomotor factors influence a person's engagement in IVR that can enhance levels of distraction, such as presence, and have been found to influence the degree of pain relief produced by an IVR environment.^53^ In this study, distraction mechanisms are leveraged by environments that are labeled as “scenic,” whereas embodiment mechanisms are leveraged by environments labeled as “somatic.”

Immersive virtual reality has the potential to encourage neuroplasticity and induce cortical reorganization, but much of this relies on the capacity of the IVR environment to create a highly immersive experience.^31,32^ Thus, although IVR appears to have some utility in treating chronic pain, the specific types of IVR environments and the psychometric properties of IVR that may promote lasting pain attenuation are largely unexplored concepts.^11^ Here, we evaluate the ability of different IVR environments in attenuating pain and examines the influence of immersion and presence in modulating pain in individuals with NP post SCI. We hypothesized that IVR environments that promote embodiment will be associated with greater NP reduction compared with those that promote distraction and that the degree of pain relief achieved by IVR environments will correlate with levels of immersion and presence.

## 2. Methods

### 2.1. Study design and setting

This pilot, double-blinded, parallel, multiarm, randomized, clinical trial investigated the feasibility of VE for treating chronic NP in people with SCI. The study was approved by Mount Sinai's Program for Protection of Human Subjects (STUDY-17-01084) and registered on ClinicalTrials.gov (Identifier: NCT04700033). Participants provided informed consent before enrollment, with sessions offered at the clinic or remotely.

### 2.2. Randomization and concealment

Participants were randomized into 2 treatment groups and 1 control group using simple randomization (1:1:1 on Excel) and stratified by injury level (paraplegia or tetraplegic). Virtual environments within each group (control, somatic, scenic) were randomized for the 12 sessions. Participants and outcome assessors were blinded to group allocation throughout the study.

### 2.3. Participants

Participants were recruited from the Mount Sinai SCI outpatient clinic or self-referred by contacting the phone number or e-mail address available in institutional review board–approved study flyers posted around the hospital, from social media advertisements, or at clinicaltrials.gov.

Participants included were adults older than 18 years, with a history of SCI (at least 6 months postinjury, with any level of injury or neurological impairment) and chronic NP below the level of injury for at least 6 months following trauma or disease of the spinal cord; presenting, with a pain intensity of at least 2 of 10 on the numerical pain rating scale (NPRS) test at baseline and stable pharmacological treatment for at least 4 weeks before the study. Participants were instructed to maintain adherence to their current treatment plans throughout the trial and refrain from altering pharmacological management. Exclusion criteria included psychiatric or neurological disorders (eg, history of stroke or severe depression), history of head injuries causing cognitive or visual impairment, severe vertigo, or visual impairment. Severe depression was screened through the Beck depression inventory (score cutoff: ≥40).

### 2.4. Data collection and outcomes

**Figure 1.**
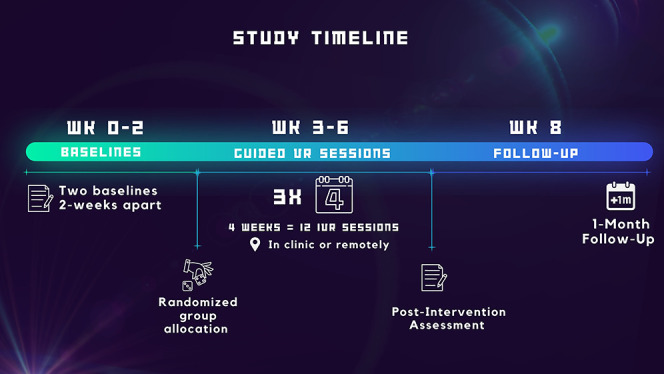
Visual illustration of study design and timeline. Weeks 1 to 2: baseline outcome measures (NRS, NPSI, NPRS, BDI, SCIM, ITQ). Weeks 3 to 6: 20 minutes VR Environment, 3x week, 4 weeks, NRS (pre/postintervention), and presence questionnaire. Postintervention assessments (NRS, NPSI, NPRS, BDI, SCIM, ITQ) were taken in the last session on week 6 and 1-month postintervention (week 12). BDI, Beck depression inventory; ITQ, immersive tendencies questionnaire; NPRS, numerical pain rating scale; NPSI, neuropathic pain symptom inventory; NRS, numerical pain scale; SCIM, spinal cord independence measure.

Baseline characteristics included age, gender, race, education, SCI etiology (traumatic or nontraumatic), time since SCI diagnosis, level of injury, tetraplegic or paraplegic, and American Spinal Injury Association Impairment Scale (ASIA) neurological classification. Participation in the study lasted 8 weeks, with 2 baseline appointments, a 4-week intervention period, a postintervention assessment, and a 4-week follow-up (Fig. 1).

### 2.5. Primary outcome measures

Neuropathic Pain Symptom Inventory (NPSI): Assesses quantitative and qualitative properties of NP. It includes 12 items, assessing spontaneous pain, brief attacks of pain, provoked pain, and abnormal sensations in the painful area. This is a validated tool for measuring changes in NP after therapeutic intervention.^8^ There are 5 subscores (burning/superficial spontaneous pain, pressing/deep spontaneous pain, paroxysmal pain, evoked pain, and paresthesia/dysesthesia) and a total score. The total score ranges from 0 to 100, with higher scores indicating greater severity. The total score was used as the primary outcome measure, assessed at baseline, postintervention follow-up, and 4-week follow-up.

### 2.6. Secondary outcome measures

(1) Neuropathic pain numerical pain scale (NRS): An 11-point scale ranging from 0 “no pain” to 10 “neuropathic pain as bad as it could be.”^24^ Assessed at baseline, pre-post session, and at the 4-week follow-up.
(2) Neuropathic pain scale (NPS): This scale includes 11 items, assessing global pain intensity, and unpleasantness, and one item that allows the patient to describe the temporal aspects of their pain and its qualities in their own words. The remaining 8 items assess specific NP qualities: “sharp,” “hot,” “dull,” “cold,” “sensitive,” “itchy,” “deep,” and “surface.” The total score is used for analysis (0–100, higher scores indicate more severity).^9,24^ Assessed at each baseline, postintervention follow-up, and four-week follow-up.
(3) Spinal cord independence measure (SCIM III): A quantitative functional outcome assessment following interventions designed to promote recovery from SCI and to increase functional achievement. It covers 19 tasks in 16 categories, with a score range 0 to 100; all activities of daily living, grouped into 4 areas of function (subscales): self-care (scored 0–20), respiration and sphincter management (0–40), mobility in room and toilet (0–10), and mobility indoors and outdoors (0–30). The total score is used for analysis (0–100, where a score of 0 defines total dependence and a score of 100 is indicative of complete independence),^2^ assessed baseline.
(4) Beck depression inventory (BDI): This is a 21-item, self-report rating inventory that measures characteristic attitudes and symptoms of depression. Total score ranges from 0 to 63; scores from 0 through 9 indicate no or minimal depression; from 10 through 18 indicate mild to moderate depression; from 19 to 29 indicate moderate to severe depression; and from 40 to 63 indicate severe depression,^57^ assessed at baseline.
(5) Immersive tendencies questionnaire (ITQ): This assesses an individual's sense of engagement and involvement in any given activity (eg, watching movies, reading books, playing video games). The total score is used for analysis (18–126, with higher scores indicating higher levels of immersion).^55^ This score was assessed at each baseline, postintervention, and four-week follow-up.
(6) UQO Presence questionnaire (PQ): Assesses presence in a specific VE by impression of being present, realness, and discomfort. The questionnaire has users rank the extent to which they agree with each question, with a total score range of 0 “not at all” to 100 “totally,”^55^ assessed after every VR session.
(7) User experience score (UES): Participants rate their experience using the technology on a scale “How much did you enjoy the environment that you just experienced?” (0–10, where 0 indicates “worst possible experience” and 10 indicates “best possible experience”). This question was created specifically for this study and assessed after every VR session.
(8) Patient's global impression of change (PGIC): Measures self-reported improvement in pain and pain-related QOL after the treatment, composed of an 11-point scale ranging from “much better” through “no change” to “much worse,” which enables participants to subjectively evaluate treatment effects, including the possibility of clinical worsening,^18^ assessed at postintervention and follow-up.

### 2.7. Virtual environments

**Figure 2.**
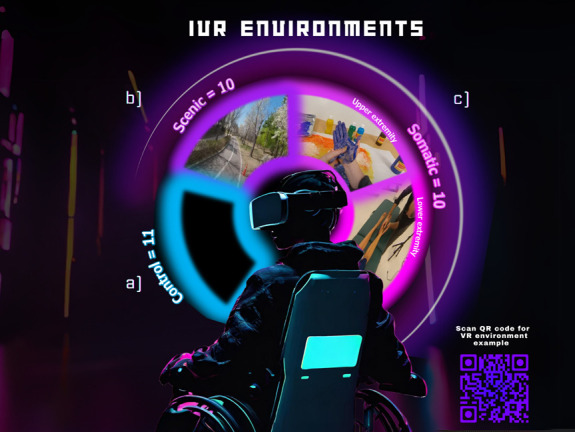
Virtual reality environmental conditions. Environments, (A) control (no video, only audio), and 2 experimental environments of (B) scenic (sceneries, no embodiment), and (C) somatic (virtual embodiment of limbs). The somatic group is composed of 2 types of VR somatic experiences, upper extremity (tetraplegia) or lower extremity (paraplegia). The 2 experimental environments are presented in a 360° view to the participants, whereas the control environment is presented with a black screen in a 360° view with audio of natural sound recordings. VR, virtual reality. Adapted from Artificial Intelligence (AI) digital rendering through Canva (Magic Studio) Prompt: “an illustrated digital character in a power wheelchair, pivoted 3/4, we see the back of their chair, as they look over their shoulder with virtual reality goggles in the dark with faded neon lighting in the background”. Pro Content license, © 2024 All Rights Reserved Canva.

Participants were presented with different VEs, one per 20-minute session, across 12 sessions over a 4-week period (3 sessions per week). The order of the VEs presented was randomized (Fig. 2).

#### 2.7.1. Scenic virtual environment

Subjects were exposed to a variety of landscape environments, presented in a 360° format in a first-person perspective, with corresponding background audio. The image would change dynamically according to the participant's head position, and virtual first-person limbs were not displayed. There were 12 different scenic environments, including a supermarket, the New York Natural History Museum, the inside of a subway car, a beach, a lakeshore, or a forest.

#### 2.7.2. Somatic virtual environment

Subjects were exposed to 360° format environments in a first-person perspective, displaying an activity performed by either upper or lower extremities with corresponding audio played concurrently. The environments focused on somatic interaction that encouraged motor imagery and subjects were instructed to embody the displayed limbs as their own. There were 24 different environments (12 for upper extremities and 12 for lower extremities). Individuals with tetraplegia were placed in somatic upper extremity videos. Individuals with paraplegia were placed in somatic lower extremity videos. Examples of upper extremity activities viewed by subjects included playing a piano, forming clay pots with a pottery wheel, performing magic tricks with playing cards, wrapping each arm with a heating pad, finger painting, and cooking. Examples of lower extremity activities viewed by subjects, included kicking and manipulating a soccer ball, sorting beans with feet, painting with feet, and kicking sand at a beach in a seated position.

#### 2.7.3. Control

Participants in the control group were presented with a solid black 360-degree screen with 12 different audio tracks of nature sounds. The tracks included rain hitting the ground, crackling fire, birdsong from multiple birds, an ocean seascape, and a forest with flowing water.

### 2.8. Headset setup

Participants were seated in their wheelchairs, and the headsets were set up independently or with the assistance of a caregiver. Two different types of headset setups were used:

(1) For at-clinic VR sessions, HTC Vive (model# OPJT100) was used. It includes 4 accessories: the headset, headphones, 2 remote controls, and 2 base station sensors. The HTC Vive was set up in a quiet and clear room free of obstructions, so participants could move freely along with the virtual environment. Before a participant's arrival, a clinical research coordinator loaded and adjusted the environment to the headset so that the environment was positioned appropriately. They then adjusted and set up the headset with headphones on the participant. The console's remote controllers were only used to set up the VR videos but not used within the virtual environment. A presession and postsession interview was conducted assessing pain, presence, and enjoyment.
(2) For at-home telehealth VR sessions, the Destek V5 headset compatible with 4.7 to 6.8 in smartphone screens was used. It is a simple, nonbattery-operated, optically designed VR device that secures a smartphone in front of the user's eyes, with buttons that manually allow a user click to navigate set up, and play and pause a video using the phone's side buttons. The headset provides a port from which users can connect their own headphones. Devices were shipped to individual's homes. Upon receipt, participants and caregivers joined a technology training call with a member of the study team. During this call, participants were provided with a curated YouTube VR playlist (displaying a personalized selection of videos, according to their group assignment), how to adjust headset and headphones, a walk through on how to load the VEs, and general instructions such as placing phone on “Non-Disturb” mode and finding a quiet and clear area free from distractions. The VR content was streamed on a secured YouTube VR link. Video access was only available during a session; videos in the playlists were blocked from viewing before and after the assigned session. All sessions were conducted over Zoom teleconferencing, on a HIPAA-secured platform.

### 2.9. Data analysis

To evaluate the effects of the study's virtual reality (VR) treatment on NP outcomes, we employed linear mixed-effects modeling. Primary and secondary outcome measures were included as dependent variables, whereas VR environment (scenic, somatic, control) and time point (baseline, post, follow-up) were included as interaction terms as independent variables. Subject ID was included as a random effect to account for variability within subjects, thus acknowledging potential correlations in repeated measures. The model was fit using the lmer function for the lme4 package (Bates et al., 2015) in R. Post hoc pairwise comparisons were calculated to interpret the interaction between VR environment and time point. Pairwise comparisons identified specific differences between time points within each VR environment. The Sidak adjustment method was applied to control for familywise error rate.^6^

Additional statistical analyses were undertaken with Stata (StataCorp, Stata Statistical Software Release: V.14). Data are presented as frequencies and proportions, or mean and 95% confidence interval (CI) where appropriate. Differences in group means were analyzed using *t*-tests, ANOVA, or repeated-measures ANOVA.

## 3. Results

**Figure 3.**
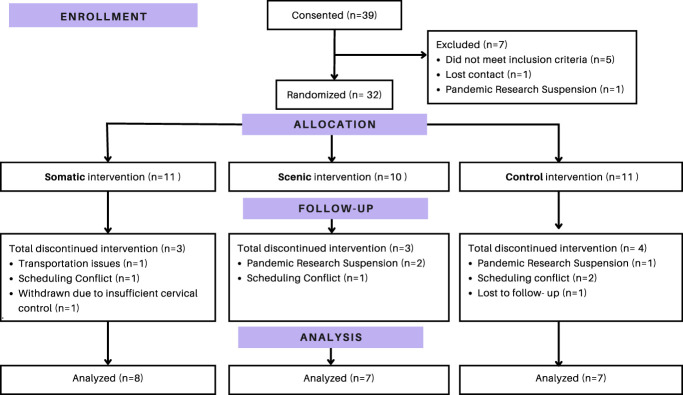
CONSORT flow diagram. CONSORT, consolidated standards of reporting trials.

Thirty-two participants enrolled in the study, with 22 (68.75%) completing the study and having data available for analysis (Table 1). Nine participants (41%) completed the study in person, with 13 (59%) completing through telehealth (Fig. 3). Demographics and SCIM, ITQ and BDI scores, at baseline are listed in Table 1 for all individuals who completed the study. All participants enrolled tolerated the intervention well. There were no adverse events (eg, severe vertigo or dizziness) reported.

**Table 1:** Participant demographics and baseline measures (n = 22).

	All participants (n = 22)	Control (n = 7)	Scenic (n = 7)	Somatic (n = 8)
Male, n (%)	15 (68)	6 (86)	4 (57)	5 (63)
Age	40 (26–61)	49 (34–66)	31 (24–41)	41 (26–61)
Race				
White	12 (55)	4 (57)	4 (57)	4 (50)
Black	5 (23)	2 (29)	1 (14)	2 (25)
Hispanic	3 (14)	1 (14)	0 (0)	2 (25)
Asian	2 (9)	0 (0)	2 (29)	0 (0)
SCI etiology, n (%)				
Nontraumatic	4 (18)	2 (29)	1 (14)	1 (13)
Traumatic	18 (82)	5 (71)	6 (86)	7 (87)
SCI category, n (%)				
Paraplegic	11 (50)	2 (29)	4 (57)	5 (63)
Tetraplegic	11 (50)	5 (71)	3 (43)	3 (37)
Injury years	12 (1–38)	15 (2–39)	9 (1–42)	13 (1–28)
ASIA score, n (%)*				
A	6 (32)	1 (20)	1 (17)	4 (50)
B	4 (21)	1 (20)	1 (17)	2 (25)
C	2 (11)	1 (20)	1 (17)	0 (0)
D	5 (26)	1 (20)	3 (50)	1 (13)
E	2 (11)	1 (20)	0 (0)	1 (13)
SCIM	63 (30–94)	63 (33–94)	69 (52–100)	57 (19–81)
ITQ	73 (43–103)	67 (43–101)	79 (60–102)	73 (37–109)
BDI	12 (2–34)	9 (2–18)	11 (4–31)	16 (0–37)

Data are presented as mean (95% confidence interval) unless otherwise stated.

* ASIA score unavailable for 3 participants (control n = 2, scenic n = 1).

ASIA, American Spinal Injury Association Scale; BDI, Beck depression inventory; ITQ, immersive tendencies questionnaire; SCI, spinal cord injury; SCIM, spinal cord independence measure.

Linear mixed-effect modeling on neuropathic pain outcome measures: NPSI, NPS, and NRS at baseline, postintervention, and follow-up assessments.

### 3.1. Primary outcome measure

#### 3.1.1. Neuropathic pain symptom inventory

Models showed a significant interaction effect for VR environment X time point (F(4,37.0) = 2.80, *P* = 0.04) as well as a significant main effect of time point (F(2, 37.0) = 5.14, *P* = 0.01). There were no significant main effects of VR environment. Post hoc pairwise comparisons of participants in the scenic VR environment demonstrated a significant within-person effect of time point, such that there was a decrease in NPSI scores from baseline to postintervention (t = 4.6, *P* = 0.0002, Cohen's D = 1.30) and from baseline to four-week follow-up (t = 2.80, *P* = 0.02, Cohen d = 0.80). Interestingly, participants in the somatic VR and control environments did not show any statistically significant within-person changes from baseline to postintervention or follow-up time points (Table 2).

**Table 2:** Neuropathic pain outcomes from baseline to postintervention and follow-up.

Outcome measure	Control			Scenic			Somatic		
	Baseline, n = 7	Postintervention, n = 7	Follow-up, n = 7	Baseline, n = 8	Postintervention, n = 8	Follow-up, n = 7	Baseline, n = 7	Postintervention, n = 7	Follow-up, n = 7
NPSI	40.43 (6.75)	39.51 (11.23)	42.54 (19.63)	51.98 (21.07)	27.98 (15.62)*	34.23 (23.25)*	50.85 (14.46)	45.44 (11.32)	45.56 (19.12)
NRS	7.36 (1.11)	5.43 (1.62)	6.29 (2.29)	5.81 (2.51)	4.00 (1.85)	3.29 (2.43)*	6.21 (1.52)	3.29 (1.70)*	4.43 (2.37)
NPS	45.21 (11.27)	41.43 (10.81)	43.57 (17.75)	44.13 (18.94)	31.63 (14.75)*	28.71 (22.51)*	48.00 (13.90)	39.14 (12.77)	42.71 (15.37)

Baselines taken before start of intervention, postintervention taken on the last session, and follow-up taken 4 weeks after the last session. Unknown, the participant missed a 4-week follow-up.

Mean (SD).

* *P* < 0.05.

NPSI, neuropathic pain symptom inventory score; NRS, pain numeric rating scale.

### 3.2. Secondary outcome measures

#### 3.2.1. Numerical pain scale

Models showed a significant main effect of time point (F(2,37.42) = 12.56, *P* = 6.7e-05). However, no statistically significant effects were observed for the VR environment or the interaction between time point and VR environment. Exploratory post hoc pairwise comparisons were conducted to gain further insights into the observed time point effect. Post hoc pairwise comparisons using Sidak adjustment revealed that the scenic VR environment exhibited a within-person effect of time point, indicating a decrease in NRS scores from baseline to follow-up (t = 3.05, *P* = 0.0123, Cohen d = 0.822). In addition, somatic VR environment exhibited within-person effect of time point, indicating a decrease in NRS scores from baseline to postintervention (t = 3.53, *P* = 0.0034, Cohen d = 1.81). There were no significant within-person effects for participants in the control environment (Table 2).

#### 3.2.2. Neuropathic pain scale

Linear mixed-effects models revealed a significant main effect of time point (F(2, 37.02) = 4.52, *P* = 0.018). However, no statistically significant effects were observed for the VR environment or the interaction between time point and VR environment. Exploratory post hoc pairwise comparisons were conducted to gain further insights into the observed time point effect. The posthoc analysis showed that participants in the scenic VR environment exhibited a within-person effect of time point, indicating a decrease in NPS scores from baseline to postintervention (t = 2.55, *P* = 0.044, Cohen d = 0.75) and from baseline to four-week follow-up (t = 2.63, *P* = 0.036, Cohen d = 0.74). By contrast, participants in the somatic VR and control environments did not show any statistically significant within-person effects of change from baseline to follow-up time points (Table 2).

### 3.3. Linear mixed-effect modeling on secondary measure of the effects of enjoyment and presence on sessional pain change score (numerical pain scale)

#### 3.3.1. Enjoyment

Models revealed a significant main effect of enjoyment (F(1, 252) = 4.68, t = −2.163, *P* = 0.03) on pain change, indicating that higher enjoyment scores are associated with a drop in pain-change scores. However, no statistically significant effects were observed for the VR environment or time point (Table 3).

**Table 3:** Sessional enjoyment, presence, and pain change scores (numeric rating scale, pre–post session).

Outcome measure	Control, n = 7	Scenic, n = 8	Somatic, n = 7
Enjoyment	6.57 (2.67)	6.19 (2.50)	7.35 (8.36)
Presence	45.79 (28.73)	53.84 (23.10)	60.79 (21.49)
Pain change (NRS)	−0.40 (0.91)	−1.11 (1.62)	−0.71 (1.68)

Mean (SD).

NRS, numeric rating scale.

#### 3.3.2. Presence

Models revealed a significant main effect of presence (F(1, 223.342) = 7.92, t = −2.8143 *P* = 0.005). This suggests that higher presence scores are associated with decreased pain-change scores. Environment and session do not significantly influence pain change in this analysis. In addition, pairwise comparisons between environments do not reveal significant differences after adjusting for multiple comparisons (Sidak) (Table 3).

### 3.4. Follow-up measures for patient's global impression of change

**Figure 4.**
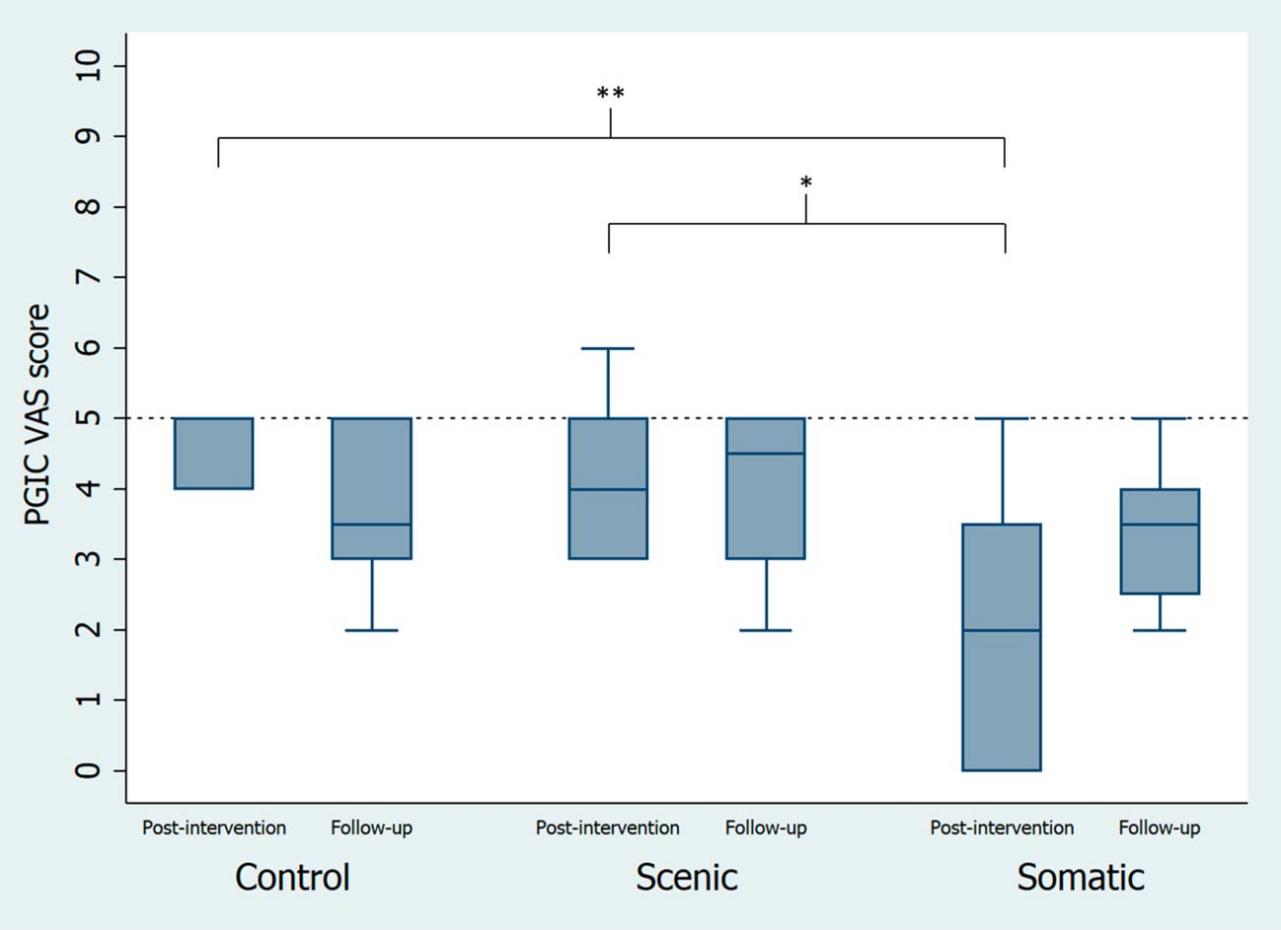
Patient's global impression of change (PGIC) visual analog scale (VAS) at postintervention and follow-up. Postintervention evaluations were taken on the last session of week 6. Follow-up evaluations were taken 4 weeks after the last session [**(*P* < 0.01), (c) *(*P* < 0.01)].

Postintervention, there was a greater impression of change observed through the PGIC VAS in the somatic group when compared with both scenic (−2 [0 to −4], *P* = 0.03) and control (−3 [−1 to −4], *P* < 0.01), with no difference observed between the scenic and control (1 [−1 to 2], *P* = 0.30), F = 8.16, *P* < 0.01 (Fig. 4). These differences were no longer present at follow-up (F = 0.49, *P* = 0.62).

There were no differences in scores on the PGIC 7-item scale across the groups at postintervention (somatic 4 [1–7], scenic 3 [1–6], control 2 [1–3], F = 1.38, *P* = 0.3) or follow-up (somatic 3 [1–5], scenic 3 [1 to −5], control 3 [1–6], F = 0.16, *P* = 0.85).

## 4. Discussion

This pilot trial evaluated the effects of IVR environments on chronic NP in individuals with SCI, comparing scenic and somatic environments. Both environments showed significant short-term pain reductions consistent with similar studies demonstrating short-term pain-relieving effects of VR therapy for individuals with SCI.^3,26,46^ Short-term pain reduction in the scenic groups, observed by primary outcome measure NPSI and secondary outcome measure NRS, suggests that participants may have benefited from some of the distraction elements of IVR, supported by other studies using distraction methods to modulate pain signaling pathways.^5,22,37^ Pain relief by distraction is theorized by gate control theory, which posits that limiting a person's attention to pain will decrease their perception of pain.^37,39,41^ These results indicate a need for further exploration of distraction mechanisms through gate control theory in NP response to VR.

This study designed somatic environments to induce neuroplastic changes related to the embodiment presented in the virtual environment. Previous work has shown that observation of movement can stimulate corticospinal excitability and a reduction in intracortical inhibition in the motor cortex, facilitating motor performance and promoting restoration of the functional cortical representation of the painful body area.^7,19,30,36,44^ In the somatic VE group, short-term reductions in pain were observed in secondary outcome measure NRS. Reversion after 4 weeks, however, suggests that the mechanism driving the analgesic effect was insufficient to induce neuroplasticity for lasting pain relief. Future studies should be designed to determine the appropriate dosage or be conducted over a longer term to address this issue. These small but significant findings may serve to highlight the need for further investigations to address the limitations observed in this study. For example, the challenging task of embodying a virtual body may contribute to the limitations encountered in testing our primary hypothesis, which posited that somatic environments would yield greater pain relief compared with other environments. In addition, it is important to recognize that short-term pain reductions are beneficial in this population. Exploring if increased or repeated exposure can extend (and potentially enhance) pain-relieving effects remains a topic worth exploring in future initiatives. Given the ease with which IVR therapies were deployed to in-home environments and the lack of adverse events in the cohort, it is also highly feasible for these therapies to be delivered to patients long-term so that they can continue to experience short-term benefits if a means to create lasting effects is not devised. Ultimately, both environments provided some type of long-term and short-term pain relief, offering multiple avenues for further investigations beyond our initial hypothesis.

Across all groups, presence and enjoyment scores were significantly associated with a reduction in pain scores postsession. Enjoyment has previously been described to positively influence motor outcomes in gamified neurorehabilitation studies, suggesting its potential relevance in pain therapies aimed at promoting neurorehabilitation.^14,47^ Notably, the relationship between presence and enjoyment of IVR environments and NP relief highlights the potential importance of tailoring IVR therapies to individual patient preferences to optimize both enjoyment and presence. Furthermore, participants randomized to the somatic group reported greater improvements in quality of life according to their PGIC VAS when compared with scenic and control groups. The PGIC VAS enables participants to subjectively assess treatment effects, including the possibility of clinical worsening, emphasizing the need for future investigations to incorporate subjective evaluations and personal experiences.^18^ This study thus presents 2 distinct and valuable approaches for evaluating pain outcomes in this population: through psychomotor variables and subjective assessments of quality of life.

A major limitation of this study was our relatively small sample size and our attrition rate due to study interruptions by the COVID-19 pandemic. This limited our ability to sensitively detect significant changes across outcome measures. Until this is reproduced in a larger sample size, results should be interpreted with caution. Other limitations pertain to the practicality of the technologies. Both the HTC Vive and Destek systems were reported to be somewhat uncomfortable to use for long periods; especially individuals with tetraplegia and limited cervical muscle strength and active range of motion. The single participant who withdrew from the study because of limited cervical range of motion was an individual living with a high cervical SCI and withdrawal from this study highlights a limitation in the universal design of head-mounted display technologies. If IVR is indeed a feasible and effective treatment for chronic NP pain in people with SCI as this work would suggest, every effort must be made for the inclusive design of lighter-weight head-mounted displays that can more easily be used by those with severe disabilities. Finally, variables that commonly influence pain outcomes should also be explored, for instance, use of medications such as opioids, anticonvulsants, and muscle relaxants.

Follow-up studies are required to confirm these findings, particularly including larger sample sizes, which may potentially be achieved with crossover designs, and intention-to-treat approaches. Further research is required to explore the appropriate dose–response dynamics of both styles of IVR environments. In addition, designing personalized IVR interventions that account for the multidimensional aspects of an individual's pain experience is a valuable area for future research, allowing for personalized IVR environments and avatars.

In conclusion, all environments were well-tolerated. Scenic VR was associated with significant, short-term reductions in NP in people with SCI across all measures of NP. Enjoyment of the IVR environment being presented, and increased feelings of presence in the IVR environment were associated with greater immediate reductions in pain, indicating the potential importance of a personalized approach for IVR therapies. This work further highlights the value of IVR as a nonpharmacological pain intervention and presents multiple avenues for future investigations.

## Disclosures

The authors have no conflict of interest to declare.
